# Loss of CtIP disturbs homologous recombination repair and sensitizes breast cancer cells to PARP inhibitors

**DOI:** 10.18632/oncotarget.6715

**Published:** 2015-12-22

**Authors:** Junhui Wang, Qianshan Ding, Hiroaki Fujimori, Akira Motegi, Yoshio Miki, Mitsuko Masutani

**Affiliations:** ^1^ Division of Chemotherapy and Clinical Cancer Research, National Cancer Center Research Institute, Tokyo 104-0045, Japan; ^2^ Department of Molecular Genetics, Division of Medical Genomics, Medical Research Institute, Tokyo Medical and Dental University, Tokyo 113-8510, Japan; ^3^ Department of Gastroenterology, Renmin Hospital of Wuhan University, Wuhan 430060, China; ^4^ Department of Radiation Genetics, Kyoto University Graduate School of Medicine, Kyoto 606-8501, Japan; ^5^ Department of Frontier Life Sciences, Nagasaki University Graduate School of Biomedical Sciences, Nagasaki 852-8588, Japan

**Keywords:** CtIP, breast cancer, PARP inhibitors, 53BP1

## Abstract

Breast cancer is one of the leading causes of death worldwide, and therefore, new and improved approaches for the treatment of breast cancer are desperately needed. CtIP (RBBP8) is a multifunctional protein that is involved in various cellular functions, including transcription, DNA replication, DNA repair and the G1 and G2 cell cycle checkpoints. CtIP plays an important role in homologous recombination repair by interacting with tumor suppressor protein BRCA1. Here, we analyzed the expression profile of CtIP by data mining using published microarray data sets. We found that *CtIP* expression is frequently decreased in breast cancer patients, and the patient group with low-expressing *CtIP* mRNA is associated with a significantly lower survival rate. The knockdown of *CtIP* in breast cancer MCF7 cells reduced Rad51 foci numbers and enhanced f H2AX foci formation after f-irradiation, suggesting that deficiency of *CtIP* decreases homologous recombination repair and delays DNA double strand break repair.

To explore the effect of CtIP on PARP inhibitor therapy for breast cancer, *CtIP*-depleted MCF7 cells were treated with PARP inhibitor olaparib (AZD2281) or veliparib (ABT-888). As in BRCA mutated cells, PARP inhibitors showed cytotoxicity to CtIP-depleted cells by preventing cells from repairing DNA damage, leading to decreased cell viability. Further, a xenograft tumor model in mice with MCF7 cells demonstrated significantly increased sensitivity towards PARP inhibition under CtIP deficiency. In summary, this study shows that low level of *CtIP* expression is associated with poor prognosis in breast cancer, and provides a rationale for establishing *CtIP* expression as a biomarker of PARP inhibitor response, and consequently offers novel therapeutic options for a significant subset of patients.

## INTRODUCTION

Breast cancer is the most frequently diagnosed cancer and one of the leading causes of cancer-associated deaths in women. Incidence rates have risen over the past 20 years in industrialized countries [1]. Therefore, the development of mechanism-based, targeted combination therapy that helps to improve disease-free survival and overall survival of breast cancer patients is still a major challenge.

Cells are continuously exposed to exogenous agents and biological processes that create DNA damage, which, if not repaired effectively and efficiently, can lead to genomic instability or cell death [2]. Poly(ADP-ribose) polymerase-1 (PARP-1) is an abundant nuclear enzyme that synthesizes the poly(ADP-ribose) polymer when activated by DNA single strand breaks (SSBs) [3]. Once single strand break (SSB) and double strand break (DSB) damage of DNA are produced, PARP-1 binds to the DNA and rapidly recruits x-ray repair complementation group 1(XRCC1) and tyrosyl DNA phosphodiesterase 1 (TDP1) to the site of damage to catalyze subsequent repair [4]. Repair of DSBs can be undertaken by two main pathways: homologous recombination (HR) repair (HRR) and non-homologous end-joining (NHEJ) repair [5]. BRCA1 and BRCA2 proteins normally function as important components of the HR pathway for the repair of DSBs. It has been reported that PARP inhibition leads to accumulation of DSBs by the failure of SSB repair and by replication fork collapse, which in turn requires HR-mediated repair [6-8]. In addition to blocking PARP catalytic activity at SSBs, PARP inhibitors can trap PARP enzyme at damaged DNA to form PARP-DNA complexes, which are more cytoxic [9]. PARP inhibition also leads to attenuation of alternative end joining repair through suppression of DNA polymerase θ recruitment [10, 11]. Cancers with *BRCA1/2* mutations are defective in HRR and are therefore hypersensitive to PARP inhibitors [7]. Following this approach, recent clinical trials for the treatment of *BRCA1/BRCA2*-deficient breast and ovarian tumors using specific inhibitors targeting PARP have been performed with the concept of ‘synthetic lethality’ [4, 6, 12, 13]. However, whether synthetic lethality is applicable to human cancers that have acquired other mutations/deletions in DNA repair genes has not been widely investigated.

Because CtIP (CtBP-interacting protein), which functions in the initial step of HRR with NBS1 and BRCA1 by acting as an end-resection enzyme to produce 3′-single stranded DNA, is known to be frequently downregulated in breast cancers as well as in other types of cancers, we focused on CtIP in this study and showed that breast cancer cells with defects in CtIP function are hypersensitive to the PARP inhibitors olaparib and veliparib. Thus, PARP inhibitors have therapeutic potential in the treatment of CtIP-deficient breast cancers, and our results might extend the concept of synthetic lethality to tumors bearing alterations in CtIP.

## RESULTS

### *CtIP* expression is frequently down-regulated in breast cancers

**Figure 1:**
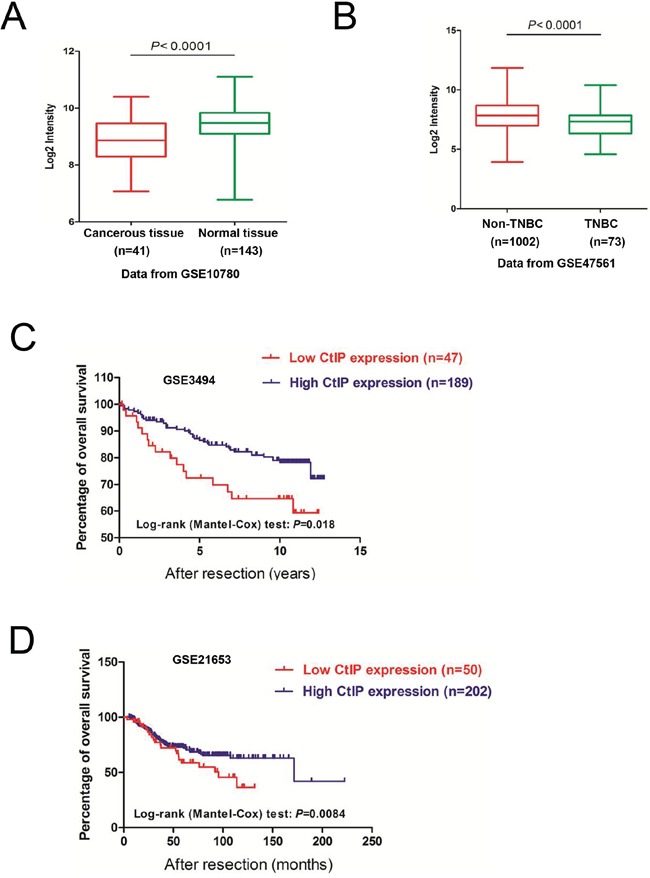
The expression of *CtIP* is decreased in human breast cancers **A.** *CtIP* mRNA expression levels are significantly reduced in breast tumors in comparison to normal breast tissues, using the publicly available microarray dataset GSE10780. *CtIP* expression is measured as log2 (probe intensities). The *P*-values were obtained from Mann-Whitney *U* or Kruskal-Wallis tests. **B.** The *CtIP* mRNA expression levels are further decreased in triple-negative breast cancers (TNBC), compared to non-triple negative breast cancers (Non-TNBC), in the publicly available microarray dataset GSE47561. *CtIP* expression is presented in log2 (probe intensities) scale. The *P*-values were obtained from Mann-Whitney *U* or Kruskal-Wallis tests. **C.** Kaplan-Meier survival curves comparing disease-free survival between cases with the lowest (≤ 20th percentile) vs. highest (> 20th percentile) *CtIP* expression (*P* = 0.018, log-rank test) (GSE3494). **D.** Kaplan-Meier survival curves comparing disease-free survival between cases with the lowest (≤ 20th percentile) vs. highest (> 20th percentile) *CtIP* expression (*P* = 0.0084, log-rank test) (GSE21653).

CtIP association with BRCA1 facilitates HRR of DSBs by initiating DNA resection [14]. Cells defective in CtIP are highly sensitive to topoisomerase I/II poisons and ionizing radiation (IR) and are unable to repair Spo11-capped meiotic DSBs [15-20]. Therefore, we investigated the relationship of *CtIP* expression in breast cancers and their clinical outcome by using two publicly available microarray datasets in the Gene Expression Omnibus (GEO) database (GSE10780 [21] and GSE3744 [21]) that contain both normal and breast cancer samples. *CtIP* expression levels were measured as log2 (probe intensities) using Affymetrix microarrays. In both these two datasets, the levels of *CtIP* mRNA in breast cancers were statistically lower than those in normal breast tissues (Fig.1A and Supplemental Fig.1A). Because patients diagnosed with triple negative breast cancer (TNBC) have a higher risk of disease relapse within 5 years than patients treated for other breast cancer subtypes [22], we compared the *CtIP* mRNA level in TNBC to that in non-TNBC by using one publicly available microarray dataset (GSE47561 [23]). We found that *CtIP* expression was significantly lower in triple negative breast cancer (Fig.1B), which is consistent with the previous study [24]. These results indicate that the expression level of *CtIP* is decreased in breast cancers. Furthermore, *CtIP* gene alterations were observed in several cancer types with either mutation, deletion or copy number variation (Supplemental Fig. 1C). However, the incidence of deletion or mutation of *CtIP* was rather low in cancers. We thus speculated that *CtIP* down-regulation might be due to epigenetic or posttranscriptional regulation.

To determine the clinical impact of reduced *CtIP* expression in human breast cancer, we assessed the association between *CtIP* mRNA levels and clinical outcome in three independent breast cancer cohorts [25-27] with clinical information (GEO database). To investigate the prognostic impact of *CtIP* expression in breast cancer, breast cancer patients were categorized into two groups based on *CtIP* mRNA expression. We found that patients with cancers displaying low *CtIP* expression levels had significantly shorter overall survival compared to those with high *CtIP* (Fig. 1C-1D, Supplemental Figure 1B). Additionally, we confirmed the correlation between *CtIP* expression and clinicopathological variables. Clinical data from GEO dataset GSE3494 was used for this analysis [25]. The samples pooled in the dataset were divided into two groups according to the expression level of *CtIP* in tumor tissue and χ 2 test was performed. As shown in Table 1, low level of *CtIP* expression was associated with p53 mutation (*P*=0.0025), PR status (*P*=0.0312), larger tumor (*P*=0.0082) and lymph nodes metastasis (*P*<0.0001). It is notable that there was an association of high *CtIP* with low PR in dataset of GSE3494, whereas no correlation was observed with ER status and information of Her2 status was not available. We calculated the data from publically available gene expression profile (GSE10780, GSE47561, GSE3494 and GSE21653). Because these datasets did not include detailed information about BRCA status, radiation therapy or chemotherapy including use of PARP inhibitor, we could not analyze the correlations between these factors and low level of *CtIP* expression. As shown in the Table 1, the low expression of *CtIP* is highly associated with lymph nodes metastasis, which could be one of the reasons for the low survival rate in *CtIP* low expression patients, although we do not know the mechanism in details. We further examined the correlation between *CtIP* expression and clinical stage in breast cancer using the data from GSE61304. Even though the expression of *CtIP* seemed to be at a lower level in higher T stage, it was not statistically significant (data not shown). Taken together, these findings suggest that *CtIP* downregulation has a critical role in overall patient survival.

**Table 1:** The correlation between *CtIP* expression and clinicopathological variables

Characteristics	No. of patients	Expression		Chi-square value	*P* value
		High	Low		
Age/year					
<55	75	62	13	0.45	0.5029
≥55	176	139	37		
p53 status					
Positive	58	47	11	0.04	0.8355
Negative	193	154	39		
p53 mutation					
Yes	72	49	23	9.15	0.0025
No	179	152	27		
ER status					
Positive	213	174	39	2.27	0.1317
Negative	34	24	10		
PR status					
Positive	190	158	32	4.64	0.0312
Negative	61	43	18		
Histologic grade					
1	67	58	9	4.94	0.0847
2	128	103	25		
3	54	38	16		
Tumor size, d/cm					
≥2	139	103	36	6.98	0.0082
<2	112	98	14		
Lymph nodes metastasis					
Yes	84	54	30	19.06	<0.0001
No	158	139	19		

Data are presented as number.

ER: Estrogen receptor; PR:Progesterone receptor.

Features of breast cancer(χ^2^ text was used), using the public expression datasets GSE3494. *CtIP* expression is measured as log2 (probe intensities).

### Loss of CtIP results in DSB repair defect

**Figure 2:**
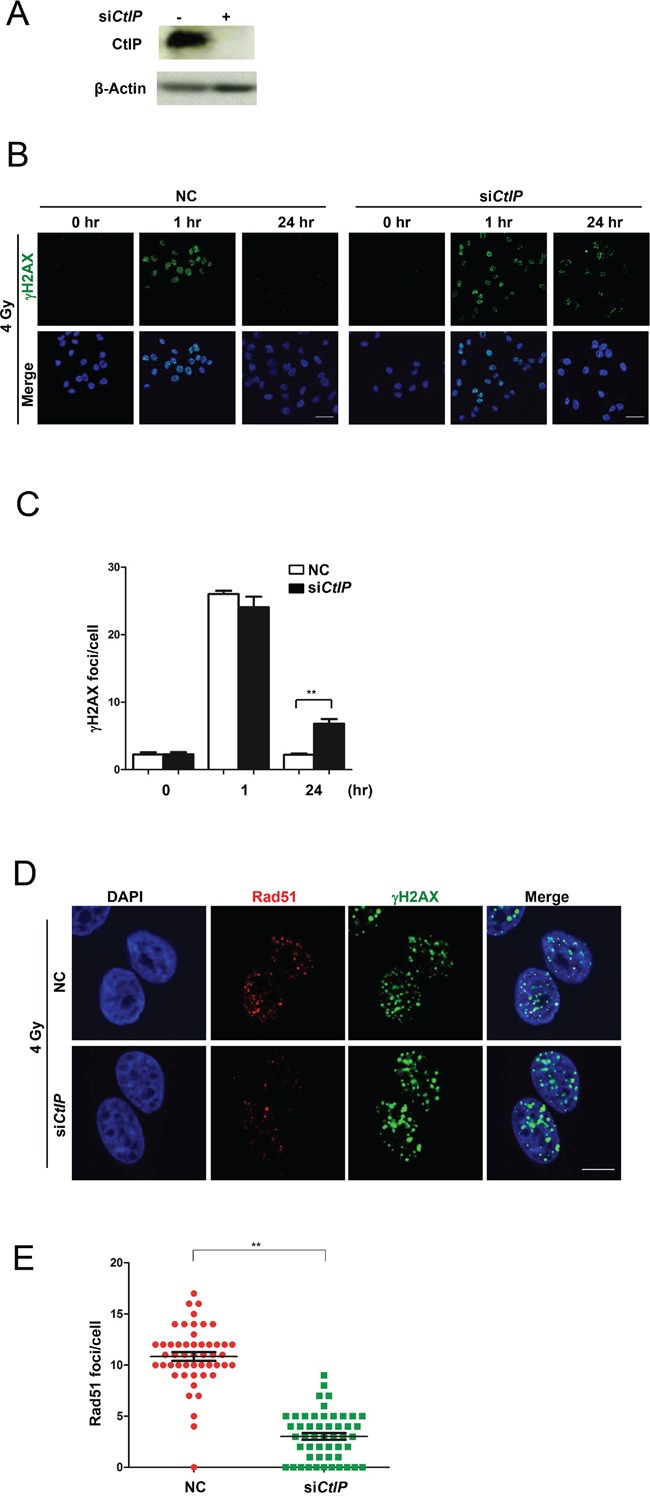
Loss of CtIP causes HRR deficiency **A.** Western blot analysis of CtIP in whole cell extracts from MCF7 cells transfected with CtIP or control siRNA (25 nM) for 48 hrs. **B.** The images of γH2AX foci after 4 Gy IR in control (NC) and CtIP-depleted MCF7 cells at different time points as indicated. Scale bar, 40 μm. **C.** Quantification of γH2AX foci in Figure 2B. Numbers of γH2AX foci were quantified from triplicated experiments (>50 cells at each condition) and are shown as mean values ± SEM. Statistical significance was calculated by one-way analysis of variance (ANOVA). ( * for *P*<0.05; ** for *P*<0.01; where not indicated, the *P* value was equal or higher than 0.05). **D.** Wild-type and *CtIP*-depleted MCF7 cells were irradiated (4 Gy) and fixed 3 hrs later. Rad51 and γH2AX foci were immunodetected with anti-Rad51 and anti-γH2AX antibodies, respectively. Cell nuclei were counterstained with DAPI. Scale bar, 10 μm. **E.** Quantification of Rad51 foci in Figure 2D. 50 cells at each condition were calculated. Mean ± SEM. Statisitcal significance, ** for *P*<0.01.

HRR-mediated DSB repair is carried out in a series of steps, the first step is nucleolytic processing, which generates 3′ single-stranded DNA (ssDNA) tails to initiate strand invasion [28, 29]. The 3′ single-stranded stretch of DNA is coated with a single-strand binding protein known as replication protein A (RPA), which is in turn displaced by RAD51 [30]. CtIP is reported to initiate 5′-strand end resection to generate 3′-overhang, which is required for the effective formation of the RPA-ssDNA complex [14]. This was further supported by a result from GSEA analysis of human breast cancers (Supplemental Fig. 2A), showing that at least 50% of the gene sets were associated with DNA damage response and repair. Detection of γH2AX has been suggested as a highly specific and sensitive marker for monitoring DSB damage and resolution [31]. Therefore, we quantified γH2AX foci formation after *CtIP* knockdown (Fig. 2A). As shown in Figure 2B and 2C, one hour after IR, the number of γH2AX foci was almost the same as at an early time point, but rather higher 24 hr later in CtIP-depleted MCF cells, when compared to control MCF7 cells, which suggested that the efficiency of DSB repair was reduced when CtIP was dysfunctional. Further, we checked HRR efficiency by checking Rad51 foci, and we found that in *CtIP*-depleted MCF cells, the number of Rad51 foci was significantly reduced 3 hrs after 4 Gy irradiation (Fig. 2D and 2E). These observations suggest that without CtIP, DNA end resection is blocked and DSBs cannot be repaired precisely and effectively by HRR.

### Loss of CtIP causes cells to be sensitive to PARP inhibitors

**Figure 3:**
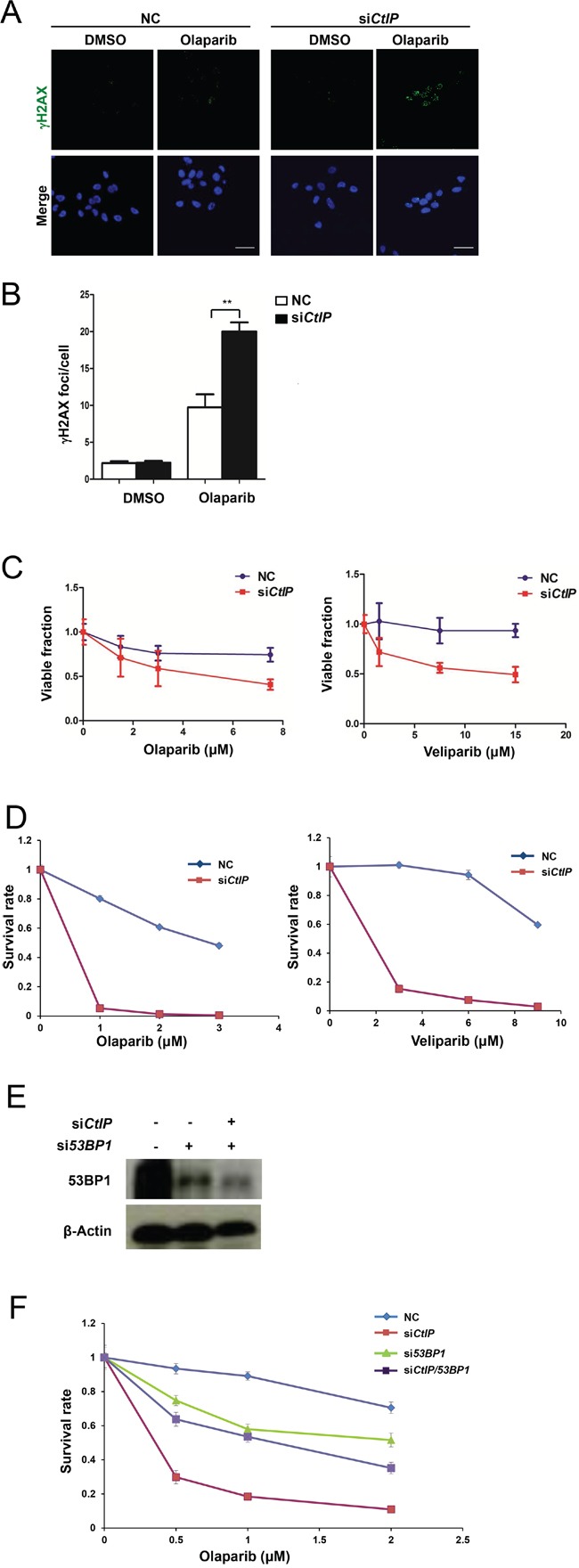
Loss of CtIP causes cells to be sensitive to PARP inhibitor **A.** PARP inhibitor showed augmented DSB DNA damage in *CtIP*-depleted MCF7 cells. Two μM of olaparib were added to wild-type MCF7 cells and *CtIP*-depleted MCF7 cells and cultured for 16 hrs. Cells were then fixed and immunostained with γH2AX antibodies. Scale bar, 10 μm. **B.** Quantification of γH2AX foci in Figure 3A. Numbers of γH2AX foci were quantified from triplicated experiments (>50 cells at each condition) and are shown as mean values ± SEM. Significance was calculated by one-way analysis of variance (ANOVA) ( * for *P*<0.05; ** for *P*<0.01; where not indicated, the *P* value was higher than 0.05). **C.** Cells were incubated with various concentrations of olaparib and veliparib, and cell viability was determined by CCK assay 96 hrs later. Plotted values are the mean values ± SEM from three independent experiments. **D.** Knockdown of *CtIP* reduces colony formation after PARP inhibitor treatment in MCF7 cells (left, olaparib; right, veliparib). Colony formation was carried out in triplicate and survival rate is calculated as mean values ± SEM. The plating efficiency of NC and si*CtIP* was 75 ± 2 % and 67 ± 2 %, respectively for the left olaparib graph. The plating efficiency of the NC and si*CtIP* was 85 ± 3 % and 60 ± 4 %, respectively for the right veliparib graph. **E.** Western blot analysis of 53BP1 in whole cell extracts from MCF7 cells transfected with *53BP1*, *53BP1*/*CtIP* or control siRNA for 48 hrs. **F.** Loss of 53BP1 rescues colony formation in *CtIP*-depleted MCF7 cells after treatment with PARP inhibitor olaparib. Plotted values are the mean values ± SEM from three independent experiments. The plating efficiency of NC, si*CtIP*, si*53BP1*, and si*CtIP/53BP1* was 95 ± 2 %, 80 ± 2 %, 46 ± 3 %, and 50 ± 4 %, respectively.

Because *CtIP*-depleted cells show HRR defect, they are expected to be more sensitive to PARP inhibitors. Here, we used two clinically used PARP inhibitors olaparib and veliparib to examine this point. The result showed that *CtIP*-depleted MCF7 cells indeed exhibited significantly increased DNA damage after treatment with these PARP inhibitors (Fig. 3A, 3B and Supplemental Fig. 3A and B), which was consistent with the recent study in ovarian cancer cells [32]. When we analyzed cell viability after treatment with olaparib and veliparib, *CtIP*-depleted cells showed decreased cell viability with MTT assay (Fig. 3C) and in colony formation assay (Fig. 3D), which was similar to *BRCA1* deficient cells (Supplemental Fig. 3D and E) [7, 33]. It was reported that in *BRCA1* deficient cancer cells, loss of 53BP1 leads to PARP inhibitor resistance [34, 35], therefore we checked whether the loss of 53BP1 can also cause PARP inhibitor resistance in *CtIP*-depleted cells. As shown in Fig. 3E and 3F, we found that loss of 53BP1 itself leads to sensitization to a PARP inhibitor, and the loss of CtIP causes cells to be highly sensitive to a PARP inhibitor, however, double loss of 53BP1 and CtIP can result in resistance to a PARP inhibitor compared to the loss of CtIP. This observation therefore substantiates the finding that loss of CtIP is associated with sensitivity towards PARP inhibition.

### CtIP loss results in increased PARP inhibitor sensitivity *in vivo*

**Figure 4:**
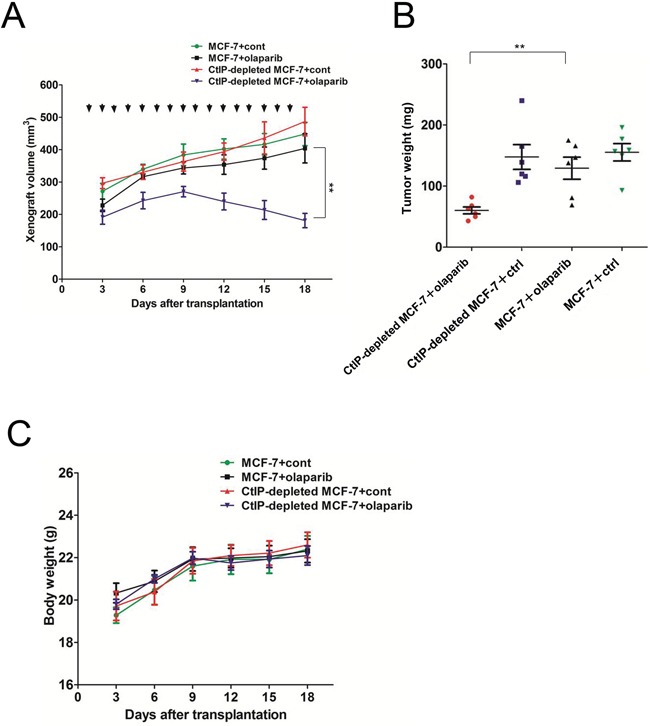
AZD2281 treatment decreased growth of MCF7 xenograft tumor **A.** Loss of CtIP significantly reduces xenograft growth after PARP inhibitor olaparib treatment. A total of 1× 10 ^7^ cells of MCF7 cells or CtIP-depleted MCF7 cells were injected into the backs of nude mice (*n* = 6 for each group). Tumor-bearing animals were intraperitoneally injected daily with olaparib (50 mg/kg) or DMSO. Xenograft size was measured every 3 days. Quantitated xenograft volume is shown (error bars represent SEM). Significance was calculated by one-way analysis of variance (ANOVA) ( * for *P*<0.05; ** for *P*<0.01). Arrows show the dates when the mice were treated with the drugs. Because tumor cells were injected with equal volume of Matrigel, which remained to be present, the xenograft volume contained that of Matrigel. **B.** Tumor weight was measured after 16 days treatment with olaparib in each cohort. Quantification of tumor weight is shown (error bars represent SEM). Significance was calculated by one-way analysis of variance (ANOVA) (* for *P*<0.05; ** for *P*<0.01). **C.** Body weight was measured every 3 days in each experimental group. There was none of the mice showing significant changes in body weight.

To assess the therapeutic effect of olaparib on *CtIP*-depleted cells *in vivo*, we investigated the ability of olaparib to suppress the growth of a *CtIP*-depleted MCF7 cell line-derived xenograft tumor. MCF7 or *CtIP*-depleted MCF7 cells were subcutaneously grafted into Balb/c nude mice. Two days after transplantation, mice were treated daily with olaparib or a vehicle. At day 3, olaparib treated two groups (siControl (black line) and si*CtIP* (violet line)) showed a slightly lower growth, compared to the group without olaparib treatment (siControl (green line) and si*CtIP* (red line)), although it was not statistically significant (Fig. 4A). This decreased growth could be due to the effect of olaparib, as we observed that the plating efficiency was slightly decreased from 75% to 67 % in the presence of olaparib as described in Fig. 3D legend. Sixteen days after treatment, olaparib caused a clearly and statistically significant inhibitory effect on tumor volume in *CtIP*-depleted MCF7 xenografts (Fig. 4A and 4B). The tumor weight was much less in olaparib-treated *CtIP*-depleted MCF7 xenografts, when compared to vehicle treatment or other groups on day 16 (Fig. 4B). During this period, we noted that in the xenograft experiment, each of the treatment regimes was equally well tolerated, with none of the mice showing significant changes in bodyweight (Fig 4C).

## DISCUSSION

In recent years, several PARP inhibitors have been developed and subjected to clinical trials for the treatment of cancer [36-38]. Using PARP inhibitors as the synthetic lethal approach represents a powerful new strategy for therapeutic intervention [39, 40]. Recently Lin et al. showed that *CtIP* knockdown sensitized ovarian cancer cells to olaparib [32]. Here, we found that *CtIP*-depleted breast cancer cells show enhanced sensitivity to PARP inhibitors olaparib and veliparib. In agreement with an underlying defect in DDR, siRNA targeting *CtIP* caused a concomitant increase and persistence in γH2AX formation and impaired DNA damage-induced Rad51 foci formation. This suggested that the cause of PARP inhibitor sensitivity in cells depleted of CtIP might involve a defect in DNA end-resection, which led to dysfunctional HR. We further showed the double loss of 53BP1 and CtIP can result in resistance to a PARP inhibitor compared to the loss of CtIP. The CtIP deficiency caused severe HRR defect probably at the initiation process, and the 53BP1 deficiency under *CtIP* knockdown condition may have partially rescued HRR but have a defect in NHEJ repair. This condition thus resulted in a slightly lower survival compared with the 53BP1 deficiency alone, where NHEJ is defective but HR is active. This observation is consistent with the notion that 53BP1 cooperating with RIF1 and PTIP promotes NHEJ repair [41] and thus its absence negatively affects the sensitivity to a PARP inhibitor in MCF-7 cells.

The human CtIP is a nuclear protein which is widely expressed in various human tissues [42]. It is phosphorylated upon DNA damage (possibly by ATM/ATR), cooperates with the Mre11-Rad50-Nbs1 complex and BRCA1 in processing DSB broken ends [14, 43, 44]. It promotes HR by initiating DSB end resection and the formation of ssDNA [30]. Finally RAD51–ssDNA nucleoprotein filament is formed to promote strand invasion and HR. In the absence of BRCA1 or CtIP, RAD51 binding to DSB sites and the HR process are interfered with, resulting in mutagenic DNA repair, genome instability, and tumorigenesis [45]. In *CtIP*-deficient mice, early embryonic lethality and shortened life span are observed with the development of multiple types of tumors [46]. Furthermore, *CtIP* deficiency was found to be associated with a significantly increased mutation rate in a screening study of colon cancers [47]. From a mutation screening of the *CtIP* gene in 89 human tumor cell lines, 5 missense and 11 silent mutations were found [48]. Meanwhile, it has been reported that CtIP protein level was frequently quite low in breast cancer cells, especially in estrogen receptor negative breast cancers [24]. Furthermore, a recent study showed that *CtIP* was also widely mutated in patients with myelodysplastic syndrome (MDS) and acute myeloid leukemia (AML) (MDS/AML) [49]. All these findings reveal that CtIP plays an important role in tumorigenesis.

In conclusion, *CtIP* is frequently downregulated in breast cancer cells and our studies revealed that the expression level of *CtIP* in breast cancer patients is correlated with overall survival. Loss of CtIP results in HRR defect, providing the rationale to investigate the clinical significance of *CtIP* and its potential use as a biomarker to the application of PARP inhibitors in breast cancer treatment.

## MATERIALS AND METHODS

### Cell lines

MCF7 cells were cultured in RPMI 1640 medium (Invitrogen) containing 10% FCS (Hyclone), 100 U/ml penicillin and 100 μg/ml streptomycin (Gibco). All cells were grown at 37°C in a humidified atmosphere with 5% CO_2_.

### Cell proliferation assay (MTT assay)

Cells were seeded in 96-well plates in triplicate at densities of 5 × 10^3^ per well. Cell proliferation was monitored at desired time points. In brief, the MTT assay was performed using a CCK-8 assay kit (Dojindo) by adding 10 μl of CCK-8 reagent for 4 hrs. Light absorbance of the solution was measured at 450 nm with a reference of 600 nm, using a microplate reader (TECAN).

### Colony formation assay

MCF7 cells were seeded in triplicate in 6-cm dishes in complete medium. After 10-14 days of growth, cells were fixed and stained with 0.1% crystal violet, and visible colonies were counted to determine cell numbers in each colony.

### Western blot analysis

Cells at 90% confluency in the 6-cm dishes were lysed in lysis buffer. Whole cell extracts were separated by 10% sodium dodecylsulfate–polyacrylamide gel electrophoresis (SDS-PAGE) and electroblotted to PVDF membrane (Bio-Rad). Membranes were incubated with appropriate primary antibodies diluted in 5% skim milk (in PBS) overnight at 4°C. The membranes were further incubated with secondary antibodies for 1 hr at room temperature. The membranes were then reacted with a chemiluminescent reagent (Millipore) and scanned using an Image Reader LAS-1000 Pro (Fuji Film).

### Immunofluorescence microscopy

For foci analysis, MCF7 cells were irradiated (4 Gy) and left to recover for different times. Cells were incubated with PARP inhibitors olaparib (Selleckchem) or veliparib (Selleckchem) where indicated. Cells were fixed with 10% formalin neutral buffer at room temperature for 15 min, permeabilized with 0.1% Triton X-100/PBS (phosphate buffered saline) on ice for 5 min, blocked with 10% FBS/PBS, and incubated with primary antibodies diluted in 10% FBS/PBS at room temperature for 1 hr. Primary antibodies were detected by anti-mouse Alexa 488 or anti-rabbit Alexa 594 secondary antibodies (Molecular Probes). Nuclei were stained by 4′,6-diamidino-2-phenylindole (DAPI). Immunofluorescence images were captured using a Fluoview FV10i microscope (Olympus).

### siRNA transfection

Transfection with dsiRNA (Integrated DNA Technologies) was carried out using Lipofectamine^®^ RNAiMAX (Invitrogen) as recommended by the manufacturers. Negative Control (DS NC1) siRNAs were used as negative controls (Integrated DNA Technologies). Human si*CtIP* target sequence is 5′-GCTAAAACAGGAACGAATCTT-3′.

### Xenograft experiments

MCF7 cells (1.0 × 10^7^) in 0.2 ml of growth medium containing 50% volume of Matrigel (BD Biosciences) were subcutaneously injected into the back of the Balb/c nude mice (Japan SLC, Inc.). Two days after transplantation, mice were treated daily with either a vehicle or 50 mg/kg bodyweight of olaparib intraperitoneally. Tumor size was measured every 3 days and calculated using the V=1/2(L × W^2^) formula. All animal studies were performed in accordance with the Guidelines for Animal Experiments of the National Cancer Center, which meet the ethical guidelines for experimental animals in Japan.

### Quantification of foci

All images were captured at identical exposures selected so as to avoid saturation at any individual focus. Intra-nuclear foci were counted by hand from confocal images. Foci from approximately 50 cells were scored for each time point in 3 independent experiments.

### GSEA

Gene Set Enrichment Analysis (GSEA) was performed by the JAVA program. Breast cancer patient gene profiling data (GSE47561) was obtained from the Gene Expression Omnibus (GEO) site. The patients were classified into two groups according to their *CtIP* expression level (top 50%: high vs. bottom 50%: low) and GSEA was carried out to assess the effects of *CtIP* expression level on various biological activities using these two classified data sets. One thousand random sample permutations were carried out and significantly enriched gene sets were identified, which produced a nominal *P*-value < 0.05 and false discovery rates (FDR) < 0.25.

### Statistical analysis

Statistical significance of differences between different groups was determined using the Student's *t*-test. The Kaplan-Meier method was used to estimate survival curves for human patients. The log-rank test and Wilcoxon test were used to compare the differences between curves. The chi-square test was applied to analyze the clinicopathological features of breast cancer. The mutation counts and fraction of copy number altered genome data for TCGA individuals were directly downloaded from the cBioPortal for Cancer Genomics (http://cbioportal.org).

## SUPPLEMENTARY FIGURES


