# Supplementary material for: Loss of CtIP disturbs homologous recombination repair and sensitizes breast cancer cells to PARP inhibitors
Source: Oncotarget. 2015 Dec 22;7(7):7701–14. doi: 10.18632/oncotarget.6715 (PMC4884948; doi:10.18632/oncotarget.6715)
Supplement: Supplementary file 1 [file oncotarget-07-7701-s001.pdf]

SUPPLEMENTARY FIGURES

A

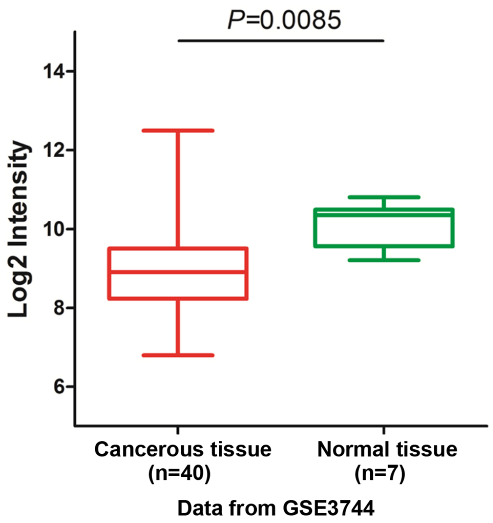

B

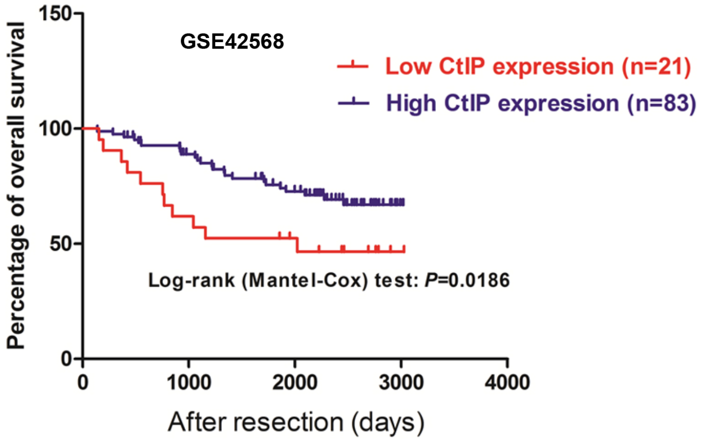

Supplementary Figure S1: A. *CtIP* mRNA expression levels are significantly reduced in breast tumors in comparison to normal breast tissues, using a publicly available microarray dataset (GSE3744). *CtIP* expression is measured as log2 (probe intensities). The *P*-values were obtained from Mann-Whitney *U* or Kruskal-Wallis tests. **B.** Kaplan-Meier survival curves comparing disease-free survival between cases with the lowest (≤ 20th percentile) vs. highest (> 20th percentile) *CtIP* expression ($P = 0.0186$, log-rank test).

(*Continued*)

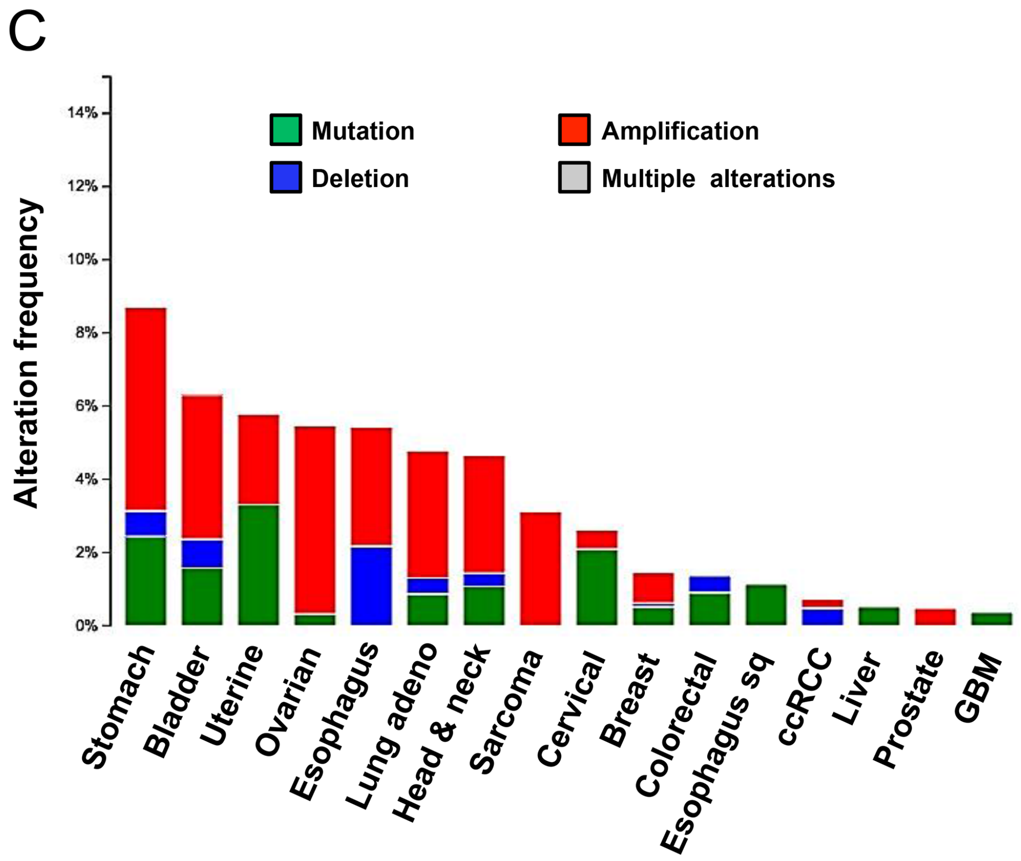

Supplementary Figure S1 (*Continued*): C. Cross-cancer summary of mutations and copy number variations of *CtIP* in several major cancers available on cBioPortal. ccRCC, clear cell renal cell carcinoma; GBM, glioblastoma.

A

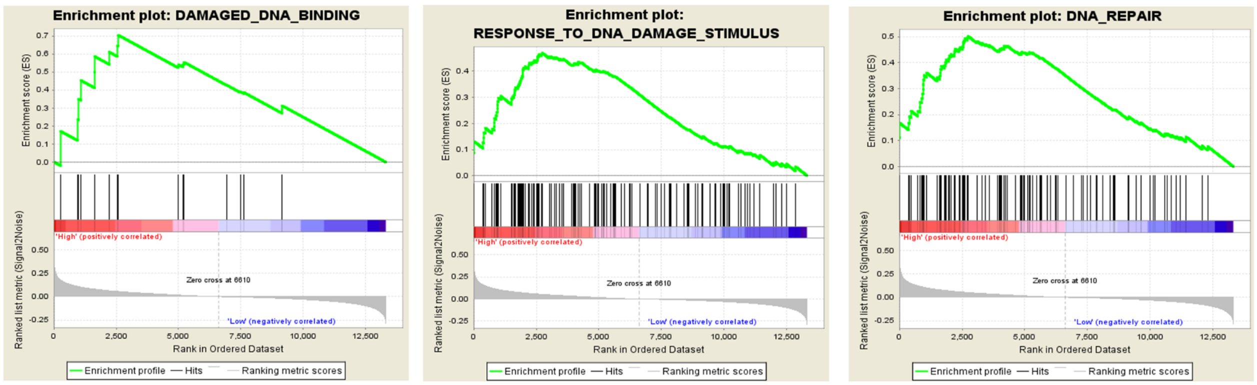

p<0.001; FDR=0.109; ES=0.703 P=0.016; FDR=0.156; ES=0.471 p=0.015; FDR=0.130; ES=0.503

B

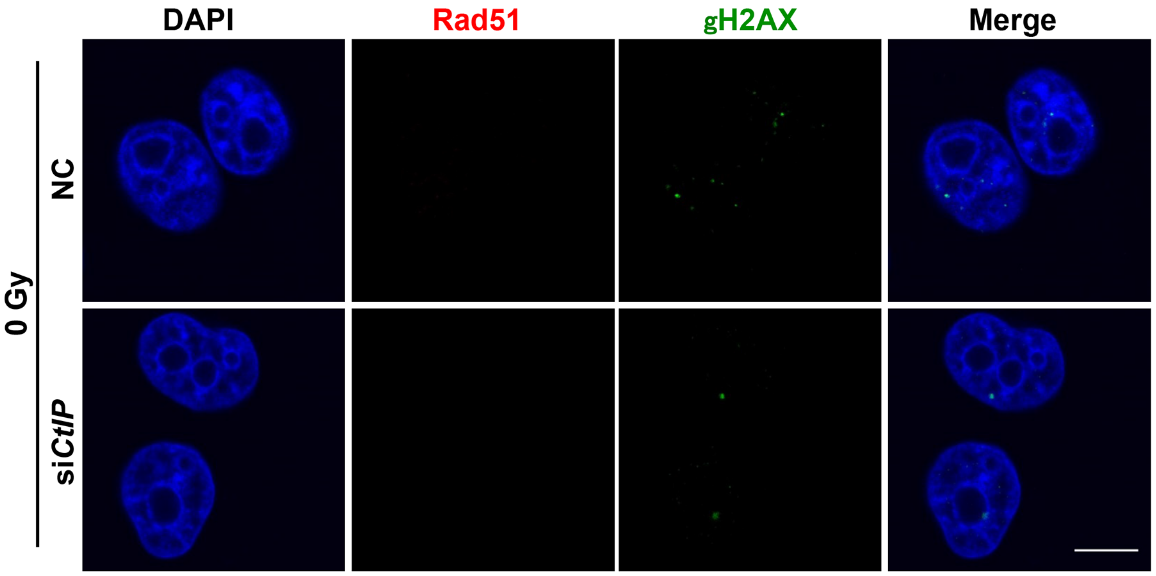

Supplementary Figure S2: A. Gene set enrichment analysis (GSEA) plot showed that there was enriched expression of gene sets involved in DNA damage and repair progression. **B.** The images of γH2AX and Rad51 foci at 0 Gy IR in control and *CtIP*-depleted MCF7 cells. Scale bar, 40 μm.

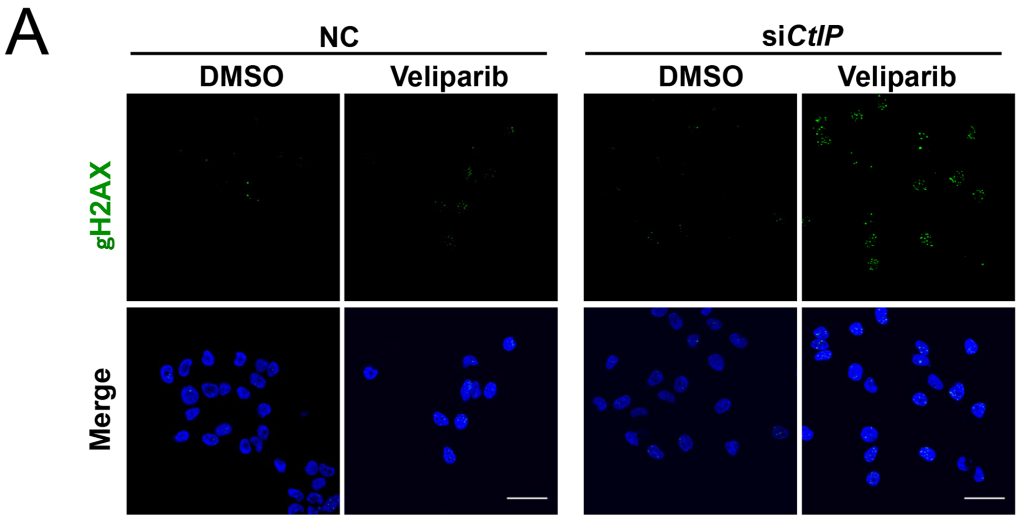

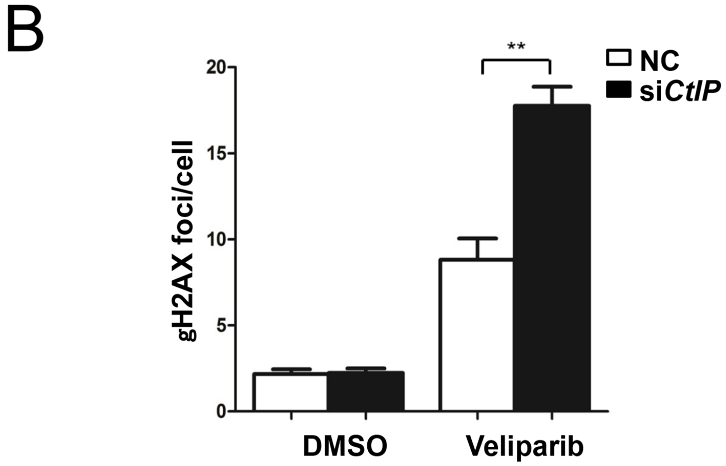

Supplemental Figure 3: A. PARP inhibitor causes more DNA damage in *CtIP*-depleted MCF7 cells. Five µM veliparib was added to wild-type MCF7 cells and *CtIP*-depleted MCF7 cells and cultured for 16 hrs. Cells were then fixed and immunostained with γH2AX antibodies. **B.** Quantification of γH2AX foci in Supplemental Figure 3A. Numbers of γH2AX foci were quantified from triplicated experiments (>50 cells at each condition) and were shown as mean values ± SEM. Significance was calculated by one-way analysis of variance (ANOVA) (* for $P<0.05$; ** for $P<0.01$; where not indicated, the P value was equal or higher than 0.05).

(*Continued*)

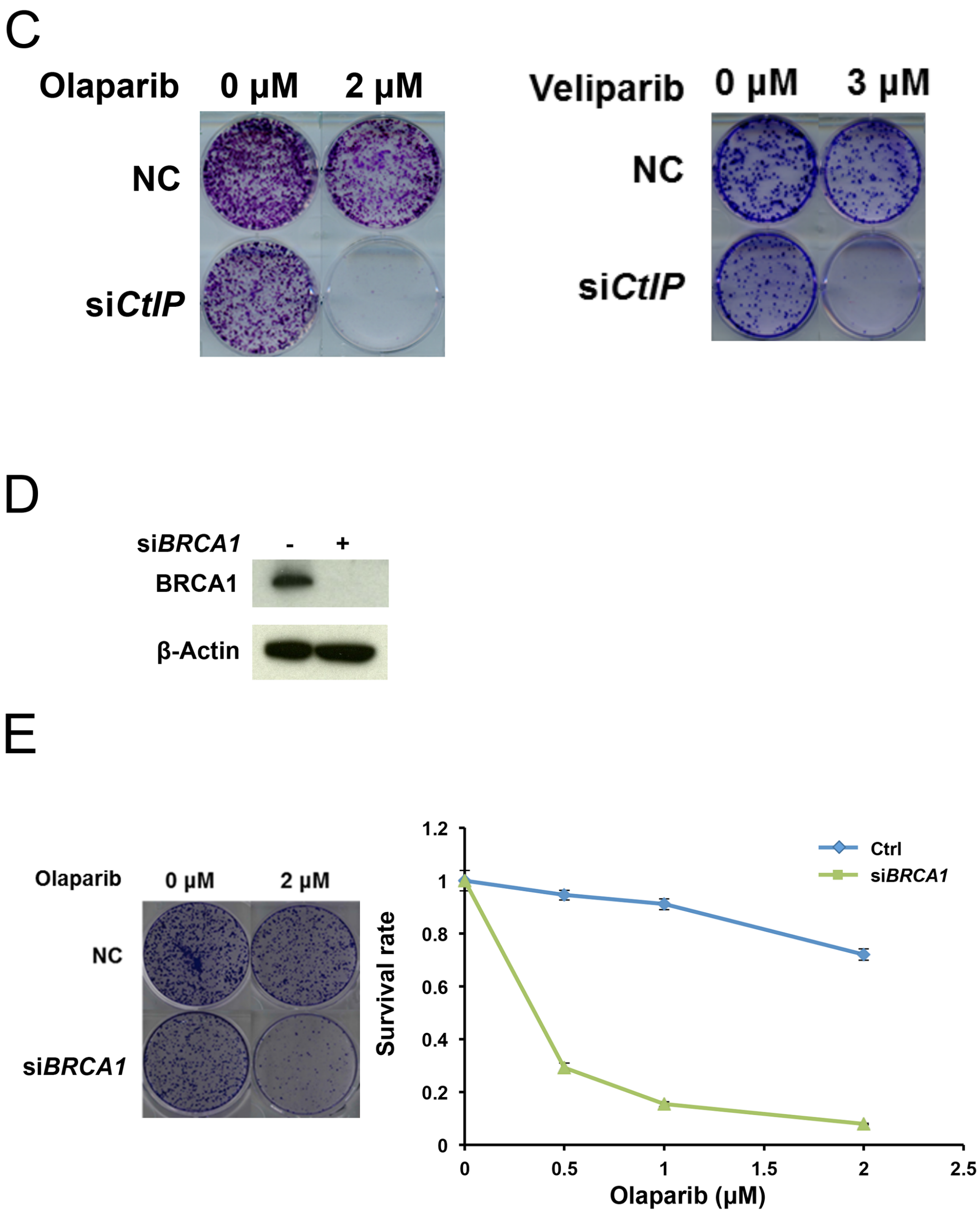

Supplementary Figure S3 (*Continued*): C. Knockdown of *CtIP* reduces colony formation after olaparib and veliparib treatment in MCF7 cells. **D.** Western blot analysis of BRCA1 in whole cell extracts from MCF7 cells transfected with *BRCA1* or control siRNA (50 nM) for 48 hrs. **E.** Knockdown of *BRCA1* reduces colony formation after PARP inhibitor treatment in MCF7 cells. The plating efficiency of NC and si*BRCA1* was 95 ± 2 % and 69 ± 3 %, respectively.
